# The impact of Hashimoto’s thyroiditis on endoscopic thyroidectomy in patients with papillary thyroid carcinoma

**DOI:** 10.3389/fonc.2025.1623966

**Published:** 2025-10-22

**Authors:** Zhen-Xin Chen, Jing-Bao Chen, Feng-Shun Pang, Zhan-Hong Lin, Jian-Hong Lin, Wen-Wan Zheng, Qiu-Ping Liu, Bo Xu, You Qin

**Affiliations:** ^1^ Department of Minimally Invasive Surgery, The Second Affiliated Hospital of Guangzhou University of Chinese Medicine (Guangdong Provincial Hospital of Chinese Medicine), Guangzhou, China; ^2^ Department of General Surgery, The First Affiliated Hospital, Jinan University, Guangzhou, Guangdong, China; ^3^ Department of Thyroid Surgery, Guangzhou First People’s Hospital, Guangzhou, Guangdong, China

**Keywords:** Hashimoto’s thyroiditis (HT), papillary thyroid carcinoma, transoral endoscopic thyroidectomy via a vestibular approach, endoscopic thyroidectomy via a chest-breast approach, outcomes

## Abstract

**Background:**

Hashimoto’s thyroiditis (HT) is often associated with papillary thyroid carcinoma (PTC) and increases the difficulty of thyroidectomy. The clinical outcomes of applying the transoral approach and the transthoracic approach—the two most widely practiced endoscopic thyroid surgery techniques—in patients with PTC complicated by HT remain unclear.

**Materials and methods:**

This study is a single-center retrospective design. Clinical data on 500 patients with PTC who underwent endoscopic thyroidectomy between January 2016 and December 2022 were collected. Patients voluntarily chose either the transoral endoscopic thyroidectomy via a vestibular approach (TOETVA) or endoscopic thyroidectomy via a chest-breast approach (ETCB), were grouped accordingly, and were further subdivided into HT and non-HT groups.

**Results:**

Of 500 patients included, 140 had HT and 360 did not. The proportion of patients with stage T1 tumors was larger in the HT group than in the non-HT group. All endoscopic thyroidectomies (202 ETCBs and 298 TOETVAs) completed successfully without conversion to open surgery. The total number of retrieved lymph nodes was larger in the HT group than in the non-HT group, but the number of positive lymph nodes was smaller. Among patients treated by ETCB, the operative time was longer and the incidence of complications (transient hypoparathyroidism and transient recurrent laryngeal nerve injury) was greater in the HT group than in the non-HT group. For patients treated by TOETVA, the operative time and incidence of complications did not differ significantly between groups.

**Conclusions:**

HT appears to be associated with less aggressive tumor characteristics. TOETVA could represent a preferable option compared with ETCB for managing PTC with concomitant HT, although further prospective studies are warranted to confirm these findings.

## Introduction

The incidence of papillary thyroid carcinoma (PTC), the most common endocrine system malignancy, has increased rapidly in recent years (1, 2). Surgery is the primary treatment for PTC, while the first endoscopic thyroid lobectomy was reported in 1997 (3). After nearly three decades of development, numerous endoscopic approaches for thyroidectomy have been reported. Among these, endoscopic thyroidectomy via a chest-breast approach (ETCB) represents the earliest and most extensively adopted technique. The first totally transoral video-assisted thyroidectomy was documented in 2009 (4) and was classified as a type of NOTES procedure. Transoral endoscopic thyroidectomy via a vestibular approach (TOETVA) completely avoids the need to make visible skin incisions and is rapidly becoming a popular alternative (5). ETCB and TOETVA are currently used in the treatment of PTC in many medical centers, but they have shortcomings. ETCB is suboptimal for central neck dissection due to obstruction by the clavicle, while TOETVA carries increased risks of infection and mental nerve injury associated with the transoral vestibular approach (6).

Hashimoto thyroiditis (HT), the most common human autoimmune disease (7), co-occurs with PTC in approximately 23% of cases (8, 9). Since it was first reported in 1955 (10), different perspectives on this relationship have emerged. Some authors have suggested that HT is a protective factor that reduces tumor aggressiveness, possibly due to the increase in inflammatory factors or lower prevalence of BRAF mutations in co-occurring PTC and HT relative to PTC alone (11–14). However, others believe that HT negatively affects the prognosis of PTC (15).

In addition, HT increases the difficulty and complication rates of thyroidectomy (16, 17), and the same may be true for minimally invasive thyroid surgery (18, 19). Common complications associated with thyroidectomy include transient/permanent recurrent laryngeal nerve (RLN) injury, transient/permanent hypoparathyroidism, mental nerve injury, severe hematoma, and infection. This study was performed to investigate the effects of HT on PTC, ETCB, and TOETVA through the analysis of intraoperative and postoperative clinical outcomes, with the aim of identifying the most appropriate minimally invasive thyroid surgery for the treatment of PTC with concomitant HT.

## Materials and methods

### Sample

Data on patients with pathologically confirmed PTC who underwent endoscopic thyroidectomy (ETCB or TOETVA) at the Guangdong Provincial Hospital of Traditional Chinese Medicine between January 2016 and December 2022 were retrospectively reviewed, including age, gender, body mass index, tumor size, blood loss, hospital stay, operative time, No. of retrieved lymph nodes, No. of retrieved positive lymph nodes, tumor T stages, and complications. Patients with lateral lymph-node or distant metastasis, poorly differentiated PTC or other thyroid malignancies, and/or histories of cancer or thyroid surgery were excluded. After receiving detailed information about ETCB and TOETVA, all patients indicated their choice of operative procedure. The patients were allocated to TOETVA and ETCB groups, and further to HT and non-HT groups according to postoperative pathological examination findings. The ipsilateral CND was performed routinely in our center. Central compartment was identified by brachiocephalic trunk artery inferiorly, carotid artery laterally, and deep layer of deep cervical fascia posteriorly. In addition, all operations done by the same team, with You Qin serving as the main surgeon. The hospital’s Institutional Review Board approved this study, and all patients provided informed consent to participation.

### Preoperative preparation

Thyroid function tests, neck ultrasound, and other necessary preoperative tests were performed for all included patients. For patients who underwent TOETVA, gargle was provided on the day before surgery and prophylactic antibiotics were administered 30 min before surgery.

### Operative procedures

#### ETCB

**Figure 1:**
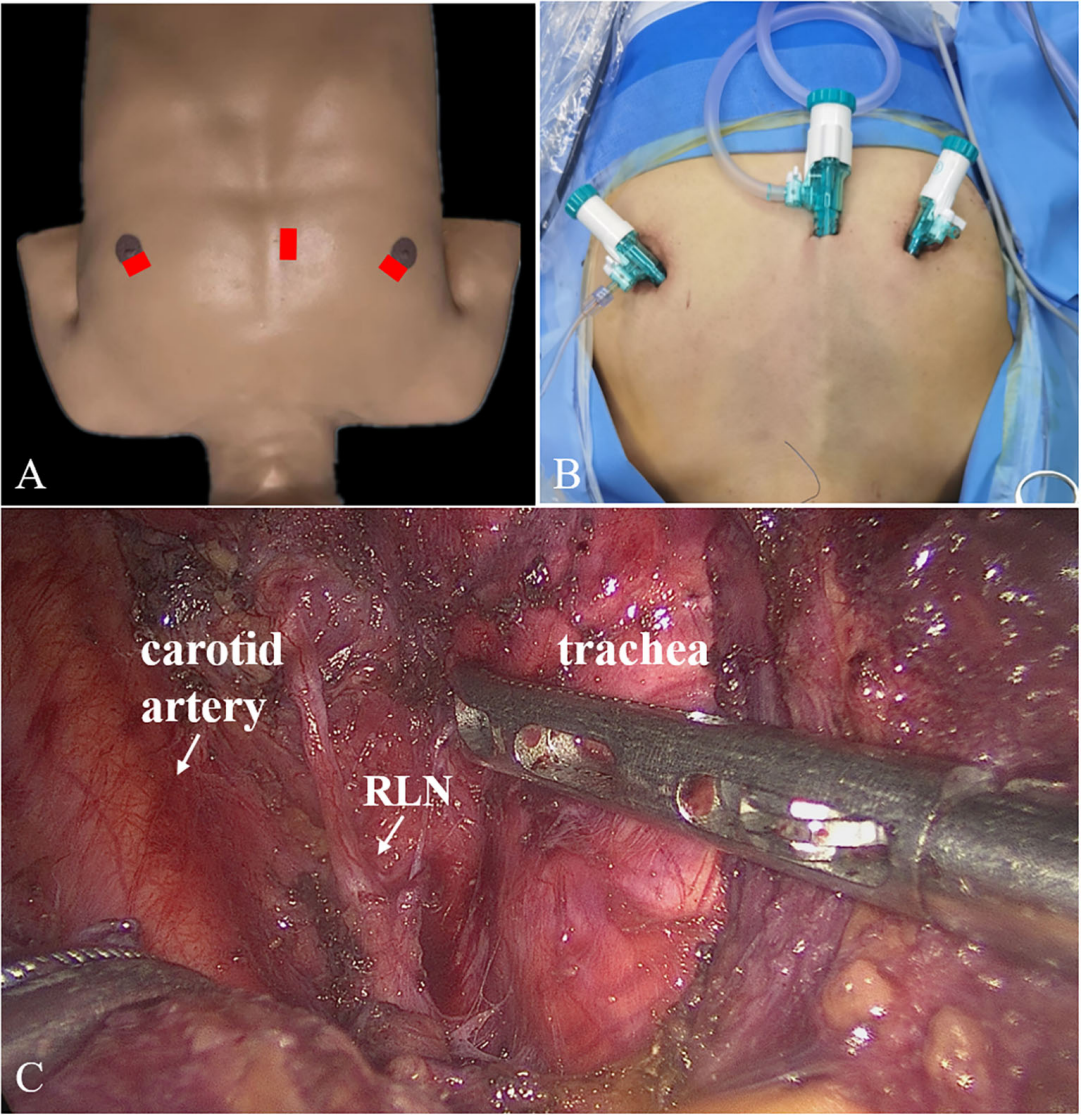
Trocars placement **(A, B)** and surgical scene **(C)** for ETCB. ETCB, endoscopic thyroidectomy via chest-breast approach.

First, a 10-mm trocar was set at nipple level next to the sternum. Two 5-mm trocars were positioned at 10–11 o’clock on the left areola and 1–2 o’clock on the right areola (**Figure 1**). Second, we established the initial working space, bounded superiorly by the larynx, inferiorly by the sternal notch, and laterally by the middle edges of the sternocleidomastoid. Third, the linea alba cervicalis was divided to reveal the thyroid, the isthmus was separated from the surface of the trachea and transected, the thyroid was retracted medially, and the carotid artery was exposed on the outside. Fourth, the upper pole of the gland was dissected and the superior thyroid vessels were ligated with the preservation of the superior laryngeal nerve and superior parathyroid gland. Fifth, the inferior thyroid vessel was ligated and the thyroid was cut close to the capsule to preserve the recurrent laryngeal nerve (RLN) and inferior parathyroid gland. Sixth, the thyroid lobe was removed completely. The contralateral thyroid lobe was also removed when necessary. Finally, the lymph nodes in the central region were dissected.

#### TOETVA

**Figure 2:**
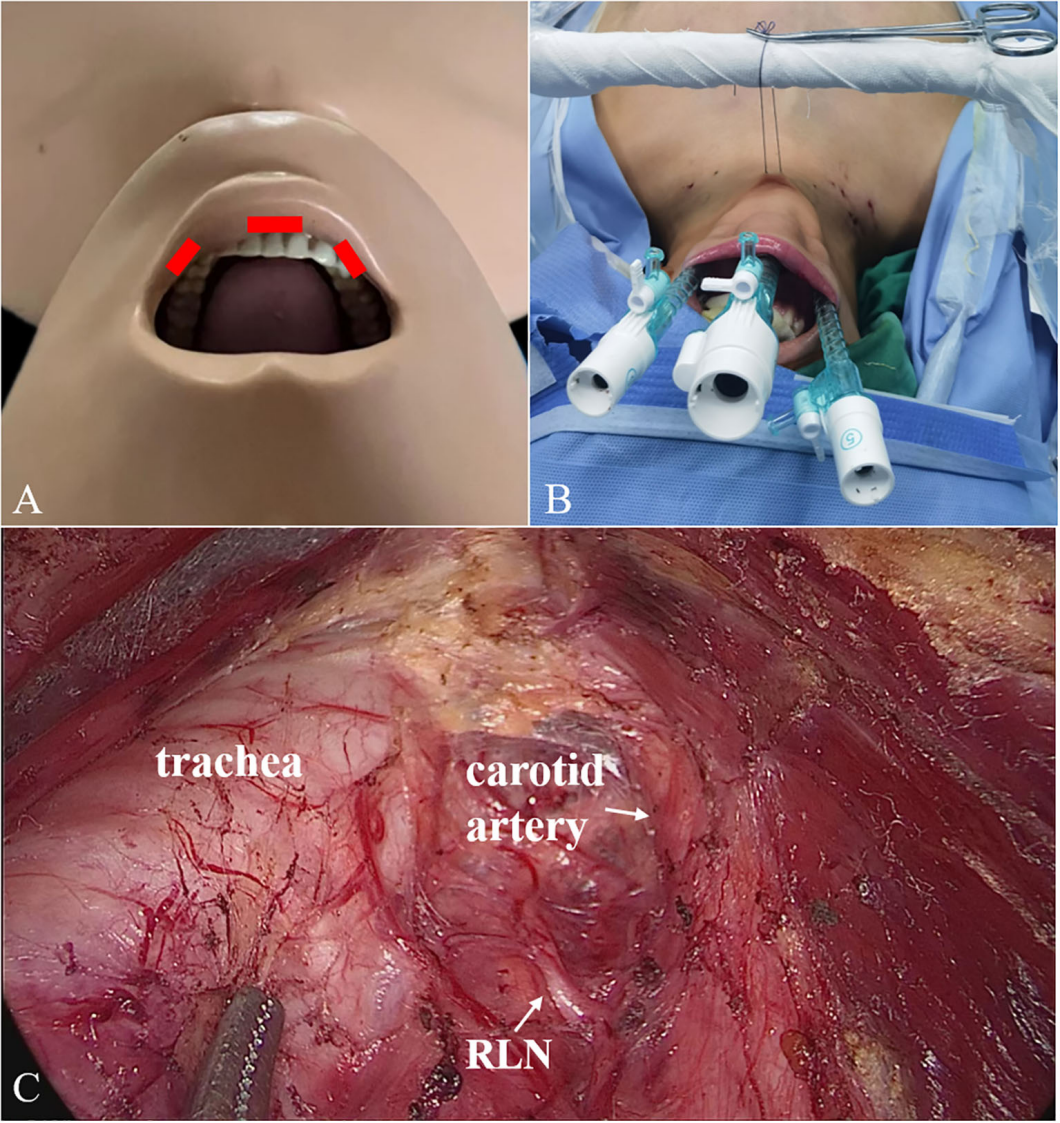
2 Trocars placement **(A, B)** and surgical scene **(C)** for TOETVA. TOETVA, transoral endoscopic thyroidectomy vestibular approach.

First, a 10-mm trocar was set in the middle of the vestibule above the inferior labial frenulum. Two 5-mm trocars were set symmetrically on the mucosa at the level of the first premolars (**Figure 2**). The second and third steps were the same as for ETCB. Fourth, the sternothyroid was partially transected to expose the superior pole of the gland, and the superior thyroid vessels were ligated. Fifth, the RLN was identified near the laryngeal entry point, and thyroid was cut close to the capsule to protect the RLN and inferior parathyroid gland. Sixth, the rest of Berry’s ligament was dissected, the inferior thyroid vessel was ligated, and the thyroid lobe was removed completely. When necessary, the contralateral thyroid lobe was also removed. Finally, CND was performed.

### Postoperative follow up

After surgery, pathology results and postoperative complications were recorded. Parathyroid hormone (PTH) levels < 11 pg/mL were considered to indicate transient hypoparathyroidism. For patients with voice impairment after surgery, laryngoscopy was regularly recommended to confirm transient RLN injury. When the PTH level and RLN injury had not recovered by 6 months after surgery, the injury was considered to be permanent. Mental nerve injury is defined as the loss of chin sensation persisting for more than six months postoperatively without recovery. Severe hematoma refers to a hematoma formed by postoperative bleeding that leads to respiratory distress and necessitates reoperation for hemostasis. Infection in this study denotes a postoperative infection that requires reoperation for debridement and drainage (**Table 1**). The patients were asked to return for evaluation by neck ultrasound every 6 months for 5 years postoperatively. Diagnostic fine needle aspiration (FNA) was performed when suspicious lesions or lymph nodes were detected.

**Table 1:** Complications and definitions.

Complications	Definition
Transient hypoparathyroidism	Parathyroid hormone levels < 11 pg/mL were considered to indicate transient hypoparathyroidism.
Permanent hypoparathyroidism	The Parathyroid hormone levels had not recovered by 6 months after surgery.
Transient RLN injury	For patients with voice impairment after surgery, laryngoscopy was regularly recommended to confirm transient RLN injury.
Permanent RLN injury	The RLN injury had not recovered by 6 months after surgery.
Mental nerve injury	Loss of sensation in the chin persisting beyond six months post-surgery without recovery.
Severe Hematoma	Postoperative bleeding led to a severe hematoma, which caused respiratory distress and required a second surgery for hemostasis.
Infection	Postoperative infection requiring reoperation for debridement and drainage.

RLN, Recurrent laryngeal nerve.

### Statistical analysis

All statistical analyses were performed with SPSS 18.0 software (SPSS Inc). Differences between groups were analyzed using the chi-squared test, chi-squared test with correction for continuity, Fisher’s exact test, and Student’s independent *t* test. Logistic regression was used for univariate analysis. All variables with *p* < 0.05 after univariate analysis were subsequently subjected to multivariate regression to determine the independence of effects. All tests were two sided, and *p* values < 0.05 were considered to be significant.

## Results

### Overall sample characteristics

In total, 500 patients with PTC (140 with and 360 without HT) were enrolled in the study. All patients underwent endoscopic thyroidectomy successfully with no conversion to open surgery. The HT group had a larger proportion of female patients, younger age, lower body mass index, and earlier tumor T stage than did the non-HT group. The total number of retrieved lymph nodes was larger in the HT group than in the non-HT group, but the number of positive lymph nodes was smaller (**Table 2**). Multivariate analysis, as shown in **Table 2**, suggested that there were several independent factors associated with the HT, including female (*p* = 0.002), more retrieved lymph nodes (*p* < 0.001), and earlier tumor T stage (*p* = 0.006).

**Table 2:** Comparison of clinical outcomes according to coexistence of HT.

Variable	With thyroiditis (n = 140)	Without thyroiditis (n = 360)	Univariate analysis p-value	OR	95%CI	Multivariate analysis p-value
Age (year)	40.6±10.7	42.9±11.5	0.035	0.987	0.968-1.006	0.182
Gender (female/male)	127/13	275/85	<0.001	2.837	1.481-5.437	0.002
BMI (kg/m^2^)	20.5±7.6	21.9±6.4	0.038	0.980	0.952-1.010	0.196
Tumor size (cm)	0.80±0.56	0.78±0.57	0.708	/	/	/
No. of retrieved lymph nodes	8.1±4.4	5.8±3.9	<0.001	1.150	1.092-1.211	<0.001
No. of retrieved positive lymph nodes	0.92±1.4	1.2±2.1	0.074	/	/	/
T1/T2+T3	125/15	287/73	0.012	2.435	1.294-4.584	0.006

HT, Hashimoto's thyroiditis; BMI, body mass index.

### ETCB group

In total, 202 patients (59 with and 143 without HT) were treated by ETCB. Operative time did not differ significantly between the HT group (135.7 ± 75.2 min) and non-HT group (117.9 ± 50.8 min) group. Besides, although the total number of retrieved lymph nodes was significantly higher in the HT group than in the non-HT group, there was no significant difference in the number of positive lymph nodes between the two groups. In the HT group, 12 patients had transient hypoparathyroidism, 7 patients had transient RLN injury, and 3 patients had permanent RLN injury. In the non-HT group, 12 patients had transient hypoparathyroidism, 2 patients had permanent hypoparathyroidism, 4 patients had transient RLN injury, 1patient had permanent RLN injury, and 1 patient had severe hematoma. The incidences of transient hypoparathyroidism (*p* = 0.017) and transient RLN injury (*p* = 0.025) were higher in the HT group than in the non-HT group (**Table 3**).

**Table 3:** Comparison of clinical outcomes according to coexistence of HT (treated by ETCB).

Variable	With thyroiditis (n = 59)	Without thyroiditis (n = 143)	Univariate analysis p-value	OR	95%CI	Multivariate analysis p-value
Operative time (min)	135.7±75.2	117.9±50.8	0.100	/		/
No. of retrieved positive lymph nodes	1.0±1.5	1.2±2.2	0.632	/		/
No. of retrieved lymph nodes	7.9±4.4	5.1±4.0	<0.001	1.168	1.076-1.268	<0.001
Age (year)	42.7±11.0	47.4±11.4	0.009	0.969	0.940-0.999	0.045
Gender (female/male)	52/7	105/38	0.022	2.512	1.000-6.310	0.050
BMI (kg/m^2^)	22.7±3.8	23.4±3.9	0.269	/		/
Tumor size (cm)	0.82±0.60	0.84±0.55	0.876	/		/
Blood loss (ml)	22.5±29.4	18.9±23.2	0.367	/		/
Hospital stay (day)	4.4±1.6	4.0±1.3	0.077	/		/
T1/T2+T3	54/5	113/30	0.033	3.988	1.303-12.200	0.015
Complications						
Transient hypoparathyroidism	47/12	131/12	0.017			
Permanent hypoparathyroidism	59/0	141/2	1.000			
Transient RLN injury	52/7	139/4	0.025			
Permanent RLN injury	56/3	142/1	0.076			
Severe Hematoma	59/0	142/1	1.000			
Infection	0	0	/			
Reoperation	59/0	142/1	1.000			
Recurrence	59/0	142/1	1.000			
Other complications^a^	0	0	/			

a Including common carotid artery injury, vagus nerve injury and so on. HT, Hashimoto's thyroiditis; ETCB, endoscopic thyroidectomy via chest-breast approach; BMI, body mass index; RLN, recurrent laryngeal nerve.

### TOETVA group

In total, 298 patients (81 with and 217 without HT) were treated by TOETVA. The OTs in the HT (117.7 ± 52.4 min) and non-HT (111.3 ± 41.5 min) groups were similar. There was also no significant difference between the two groups regarding positive lymph nodes; however, the total number of retrieved lymph nodes was significantly higher in the HT group. In the HT group, 17 patients had transient hypoparathyroidism and 3 patients had transient RLN injury. In the non-HT group, 28 patients had transient hypoparathyroidism, 2 patients had permanent hypoparathyroidism, 3 patients had transient RLN injury, 1 patient had permanent RLN injury, and 1 patient had severe hematoma. The incidences of postoperative complications did not differ between groups (**Table 4**). Notably, all patients who underwent the TOETVA were completely free from mental nerve injury and infection.

**Table 4:** Comparison of clinical outcomes according to coexistence of HT (treated by TOETVA).

Variable	With thyroiditis (n = 81)	Without thyroiditis (n = 217)	Univariate analysis p-value	OR	95%CI	Multivariate analysis p-value
Operative time (min)	117.7±52.4	111.3±41.5	0.272	/		/
No. of retrieved positive lymph nodes	0.85±1.4	1.3±2.1	0.057	/		/
No. of retrieved lymph nodes	8.2±4.4	6.2±3.7	<0.001	1.139	1.066-1.218	<0.001
Age (year)	39.0±10.2	40.0±10.5	0.441	/		/
Gender (female/male)	75/6	170/47	0.004	3.811	1.523-9.537	0.004
BMI (kg/m^2^)	18.9±9.1	20.9±7.5	0.269	/		/
Tumor size (cm)	0.78±0.53	0.74±0.59	0.570	/		/
Blood loss (ml)	16.9±14.0	15.7±14.1	0.504	/		/
Hospital stay (day)	3.7±1.1	3.5±1.0	0.112	/		/
T1/T2+T3	71/10	174/43	0.134	/		/
Complications						
Transient hypoparathyroidism	64/17	189/28	0.083			
Permanent hypoparathyroidism	81/0	215/2	1.000			
Transient RLN injury	78/3	214/3	0.350			
Permanent RLN injury	81/0	216/1	1.000			
Mental nerve injury	0	0	/			
Severe Hematoma	81/0	216/1	1.000			
Infection	0	0	/			
Reoperation	81/0	216/1	1.000			
Recurrence	81/0	215/2	1.000			
Other complications^a^	0	0	/			

a Including common carotid artery injury, vagus nerve injury and so on. HT, Hashimoto's thyroiditis; TOETVA, transoral endoscopic thyroidectomy vestibular approach; BMI, body mass index; RLN, recurrent laryngeal nerve.

## Discussion

The relationship between PTC and HT is a matter of debate (20, 21). Different rates of the coexistence of HT and PTC have been reported. The reported coexistence rate of HT and PTC from epidemiological studies is approximately 23% on average, ranging from 5% to 85% (8, 9). In this study, 28% of patients with PTC had HT. Second, no consensus on the effect of HT on the prognosis of PTC has been reached. In this study, a larger proportion of patients in the HT group had earlier tumor T stages, suggesting that HT is a protective factor for PTC. This inference is consistent with the conclusions of most researchers (22, 23). Third, the effect of HT on lymph-node metastasis is controversial. In some studies, lymph-node metastasis was more common in the presence of concomitant HT (15, 24), which may be related to increased programmed death ligand-1 (PD-L1) levels (25). In contrast, the frequency of lymph-node metastasis was lower in patients with HT than in those without HT in other studies (26, 27), which may be due to decreased PD-L1 levels caused by major histocompatibility complex class I expression in HT (13). In this study, despite a higher total retrieved lymph nodes in the HT group, the number of positive nodes was lower, indicating a lower lymph node positivity rate (*p* < 0.001). This suggests that HT may help inhibit lymph node metastasis.

Little research has been performed to examine the application of endoscopic thyroidectomy for patients with PTC and concomitant HT (28, 29), and the sample for this study is the largest examined to date. In contrast to the approach taken in previous studies, we investigated the effects of HT on ETCB and TOETVA outcomes separately. For ETCB, HT may lead to OT prolongation (135.7 ± 75.2 min vs. 117.9 ± 50.8 min) and increased complication rates. Diffuse enlargement of the thyroid gland and the formation of dense fibrotic adhesions may have adverse effects on the performance of this surgery (16, 28). For TOETVA, the OT and complication rates did not differ between the HT and non-HT groups. We attribute this lack of difference to several factors. First, all patients included in this study underwent central lymph-node dissection, and TOETVA has been suggested to be more suitable than ETCB for CND (6). The obstruction of the clavicle increases the difficulty of CND in ETCB, which may lead to increased OTs and complication rates. Second, our previous study indicated that TOETVA aids the localization and protection of the RLN and superior parathyroid gland (30).

It is noteworthy that no complications of mental nerve injury or infection occurred in the TOETVA group. Given that the reported incidence of these complications is also very low in other literature on the TOETVA (5, 31), we believe that the risks of these two specific complications can likely be effectively controlled through standardized prophylactic antibiotic use and trocar placement.

In recent years, the transoral endoscopic thyroidectomy submental vestibular approach (TOETSMVA) has been proposed and demonstrated advantages compared to the TOETVA (32). The outcomes of applying this technique in patients complicated with HT will be a focus of our subsequent attention. Additionally, postoperative thyroid dysfunction in patients with HT undergoing thyroid lobectomy has attracted attention, with the study reporting a new disease entity—painless thyroiditis (33). The incidence of thyroid dysfunction in patients with HT undergoing endoscopic thyroid lobectomy is also one of the issues we intend to investigate in the future.

The main limitation of this study is its single-center retrospective design. Multicenter prospective studies, research on underlying mechanisms, and controlled trials examining the impacts of HT on PTC are urgently needed. Second, the short follow-up time of enrolled patients has resulted in recurrence data of limited meaningfulness and a lack of survival data. Third, the small number of specific complication events limits the strength of the conclusions in the complication analysis. Fourth, our study, along with others, has demonstrated that age and gender can influence the outcomes, potentially introducing confounding and bias into the analysis (34). Besides, the six-year duration of this study may have introduced learning curve effects, which could potentially influence both operative time and complication rates. In addition, the choice of surgical approach (TOETVA vs ETCB) was made by patients rather than randomized, which may have led to systematic differences between groups. The main strength of the study is that we enrolled a large number of cases who underwent endoscopic thyroidectomies performed by the same surgical team, minimizing operator-related differences.

In summary, this study suggested that PTC with concomitant HT appears to be associated with indolent tumor characteristics. TOETVA may be safer and more effective than ETCB in the treatment of co-occurring PTC and HT.

## Data Availability

The raw data supporting the conclusions of this article will be made available by the authors, without undue reservation.
